# Supplementary material for: Effect of easing lockdown and restriction measures on COVID-19 epidemic projection: A case study of Saudi Arabia
Source: PLoS One. 2021 Sep 9;16(9):e0256958. doi: 10.1371/journal.pone.0256958 (PMC8428777; doi:10.1371/journal.pone.0256958)
Supplement: Attachment — Submitted filename: Reviewers comments.docx [file pone.0256958.s001.docx]

Responses on the reviewers’ comments

Title: Effect of easing lockdown and restriction measures on COVID-19 epidemic projection: a case study of Saudi Arabia

(PONE-D-21-01867)

Reviewer 1:

| Comments | Response |
| --- | --- |
| 1. Equation (1) and (2) is not correct. A factor of 1/N is missing. | Well spotted. Corrected. Page no 4 |
| 2. The basic reproduction number Ro is not correctly defined ("So" mentioned in Ro's definition should be the initial susceptible number. This is not defined in the manuscript either.). The proper definition should be defined by So/N, not just So. The effective reproduction number Rt is not defined nor properly referenced. | This part is thoroughly revised to reduce the confusion. Ro and Rt are defined |
| Similar to point 2, there are several parameters/terminologies in Table 1 are not defined. | Definition is provided. A note is also added |
| All figures have no captions. | Captions added for all figures |
| Ref. 15 and 17 are missing. | Added |
| 6. Results of time series are shown only in Fig.6, and the fittings are not as good as SIR ones. What's the point of using time series analysis? | Yes. As we applied both SIR and Time Series model, we plotted both lines in the chart |
| 7. Equation of Gompertz curve is mentioned on page 5, but the parameters for obtaining the fitting in the Fig.6 are not mentioned in the manuscript. | Non-linear regression is used to estimate the parameter in equation (4). Then we estimated predicted cases using parameters derived from the regression. The regression tables are shown the appendix (page 16) |
| 8. The comparison with I and R are not shown. Fig.1-6 are just daily new cases=-dS/dt and/or infected=N-S. | Yes, that is true. As we used both models to estimate *I*_t_, we focused on that only. |
| 9. The negative impact on the pandemic for early lifting of the lockdown seems obvious even without mathematical modeling. How can this work help identify the proper timing for easing the lockdown? | It is true. But when KSA lifted lockdown then Rt was less than 1. Generally, it is assumed that when Rt goes below 1, it is safe to reduce the strict measures. We showed that it is not necessarily the case. |

Reviewer 2:

| Comments | Response |
| --- | --- |
| This interesting and thought-provoking paper addresses a complex issue which is bounded by uncertainty. It is helpful to have a perspective from Saudi Arabia on this global issue. | Thank you |
| The major thing that needs to be addressed is that the results section narrative needs to have much more detail in relation to what is shown in the figures. | Exposition of the results sections have been changed providing more detail about the parameters |
| There are also economic considerations which have to be factored in as described in: Miles DK, Stedman M, Heald AH. "Stay at Home, Protect the National Health Service, Save Lives": A cost benefit analysis of the lockdown in the United Kingdom. Int J Clin Pract. 2020 Please can this cost benefit analysis be factored in | Thank you. Yes, this article is cited and a discussion on cost-benefit issue is highlighter in the discussion section |
