# Supplementary material for: Effect of easing lockdown and restriction measures on COVID-19 epidemic projection: A case study of Saudi Arabia
Source: PLoS One. 2021 Sep 9;16(9):e0256958. doi: 10.1371/journal.pone.0256958 (PMC8428777; doi:10.1371/journal.pone.0256958)
Supplement: Attachment — Submitted filename: Response to Reviewers Comments_2nd Revision.docx [file pone.0256958.s002.docx]

**Response to Reviewers’ Comments**

**Manuscript Title:** **Effect of easing lockdown and restriction measures on COVID-19 epidemic projection: a case study of Saudi Arabia (PONE-D-21-01867)**

Comment:

1. Though, the introduction looks well caved, there are more to be done to make the paper suit publication in a journal of the like of PLOS ONE. First, the authors need to be more factual and coherent in the presentation of their arguments. For instance, in making reference to the historical trend in COVID-19 outbreak globally, reference was given to January 18, 2021 while similar instance in the case of KSA referred to October 02, 2020. There is need to either update KS to 2021 or refer global data to October, 2020. The former suggestion is however preferred. See page 3 paragraph 1 lines 1-4.

Response: Revised and updated the KSA data to January 18, 2021.

Comment:

2. More so, the authors need to strengthen the contribution of their work to the literature because I am aware a number of papers have been published on the topic and in KSA in particular. The arguments that the study compared several predictions using the SIR model before and after lockdown measures are the enough.

Response: Thank you for providing suggestions for more articles. Literature review part is updated.

Comment

3. The paper in its present form is deficient in terms of contribution to the literature due to its lack deep review in recent studies relating to lockdown. The following studies should be considered.
   https://doi.org/10.1016/j.imu.2020.100420
   https://doi.org/10.1016/j.hlpt.2020.09.004
   https://doi.org/10.1007/s11869-020-00918-3
   https://doi.org/10.1007/s41996-020-00070-1
   https://doi.org/10.1080/00036846.2020.1828809
   https://doi.org/10.1016/j.trip.2020.100217

Response: All the literatures mentioned here are very relevant and hence included in the literature review. Thank you for suggesting these references.

Comment:
4. For robustness sake, the study can dwell more on lockdown data available at Google mobility data (https://www.google.com/covid19/mobility/). The data shows how visits to places, such as grocery stores and parks, are changing in each geographic region during the lockdown periods.

Response: good suggestions. However, it is beyond the scope of the current exercise. However, the limitation is acknowledged in the discussion section.

Comment

5. The language structure of the paper can be improved. For instance, the sentence structure in paragraph 2, lines 4-11 is too long. Also, when introducing new concept, full meaning should be provided before abbreviating. For instance, ICU used in paragraph 2 line 12 should be corrected and other related ones.

Response: Revised as suggested. Long sentence has been broken down to two sentences. Details on ICU is provided before using it as abbreviation.
